# Interindividual variability in sweat electrolyte concentration in marathoners

**DOI:** 10.1186/s12970-016-0141-z

**Published:** 2016-07-29

**Authors:** Beatriz Lara, César Gallo-Salazar, Carlos Puente, Francisco Areces, Juan José Salinero, Juan Del Coso

**Affiliations:** Camilo José Cela University Exercise Physiology Laboratory, C/ Castillo de Alarcon, 49, Villafranca del Castillo, 28692 Spain

**Keywords:** Exercise, Fluid balance, Hyponatremia, Athletes, Osmolality, Sex

## Abstract

**Background:**

Sodium (Na^+^) intake during exercise aims to replace the Na^+^ lost by sweat to avoid electrolyte imbalances, especially in endurance disciplines. However, Na^+^ needs can be very different among individuals because of the great inter-individual variability in sweat electrolyte concentration. The aim of this investigation was to determine sweat electrolyte concentration in a large group of marathoners.

**Methods:**

A total of 157 experienced runners (141 men and 16 women) completed a marathon race (24.4 ± 3.6 °C and 27.7 ± 4.8 % of humidity). During the race, sweat samples were collected by using sweat patches placed on the runners’ forearms. Sweat electrolyte concentration was measured by using photoelectric flame photometry.

**Results:**

As a group, sweat Na^+^ concentration was 42.9 ± 18.7 mmol·L^−1^ (minimal-maximal value = 7.0–95.5 mmol·L^−1^), sweat Cl^−^ concentration was 32.2 ± 15.6 mmol·L^−1^ (7.3–90.6 mmol·L^−1^) and sweat K^+^ concentration was 6.0 ± 0.9 mmol·L^−1^ (3.1–8.0 mmol·L^−1^). Women presented lower sweat Na^+^ (33.9 ± 12.1 vs 44.0 ± 19.1 mmol·L^−1^; *P* = 0.04) and sweat Cl^−^ concentrations (22.9 ± 10.5 vs 33.2 ± 15.8 mmol·L^−1^; *P* = 0.01) than men. A 20 % of individuals presented a sweat Na^+^ concentration higher than 60 mmol·L^−1^ while this threshold was not surpassed by any female marathoner. Sweat electrolyte concentration did not correlate to sweat rate, age, body characteristics, experience or training. Although there was a significant correlation between sweat Na^+^ concentration and running pace (r = 0.18; *P* = 0.03), this association was weak to interpret that sweat Na^+^ concentration increased with running pace.

**Conclusions:**

The inter-individual variability in sweat electrolyte concentration was not explained by any individual characteristics except for individual running pace and sex. An important portion (20 %) of marathoners might need special sodium intake recommendations due to their high sweat salt losses.

## Background

The aim of salt intake during exercise is to partially replace the amount of sodium (Na^+^) and chloride (Cl^−^) lost by thermoregulatory sweat, in order to enhance the maintenance of body water and electrolyte homeostasis [1] and to ameliorate physical and muscle performance decrements [2]. Salt intake is important for a myriad of sport modalities and training routines but it is even more relevant for endurance sports where large exercise times can trigger the occurrence of excessive fluid and electrolyte losses by sweating [3].

To estimate the requirements of salt during exercise, several investigations have been conducted to assess sweat Na^+^ concentrations during different exercise and sport activities. As a range, sweat Na^+^ concentration is 38–53 mmol·L^−1^ in football [4, 5], 20–62 mmol·L^−1^ in soccer [6, 7], 43–65 mmol·L^−1^ in swimming [8, 9], 34–38 mmol·L^−1^ in handball [10, 11], 54–73 mmol·L^−1^ in ice hockey [12, 13], 17–73 mmol·L^−1^ in marathon [14] and 46–48 mmol·L^−1^ in triathlon [1]. A recent retrospective analysis with 506 athletes [15] has determined that sweat Na^+^ concentration during exercise might vary from 13 to 105 mmol·L^−1^ but these data were obtained from athletes of very different sport disciplines (American football, baseball, basketball, soccer, tennis, cycling, running and triathlon) in very diverse weather conditions (15 to 50 °C of dry temperature). Despite all these investigations having used similar methodologies to obtain sweat samples (e.g., one or several sweat patches to collect local sweat) and similar laboratory analysis techniques (ion selective analysis or flame photometry), the outcomes of these investigations indicate a high variability in sweat Na^+^ concentration and sweat Na^+^ losses among individuals and among sports.

It is well known that sweat rate [16], ambient temperature [17], acclimatization [18] training status [19] and even the genotype of the cystic fibrosis transmembrane conductance regulator (CFTR) gene [20] can affect electrolyte absorption thru the sweat gland’s duct and thus produce significant variations in the final electrolyte concentration of thermoregulatory sweat. However, these characteristics do not fully explain the high inter-individual variance found in these previous investigations. Perhaps, the relatively small sample sizes used in these studies (number of participants between 10 and 55) [1, 4–14] have limited the possibility of obtaining explanations for the variation of sweat Na^+^ concentration during exercise.

Despite athletes of endurance disciplines being more prone to suffer water and electrolyte imbalances than athletes of shorter disciplines or team sports players [3, 21, 22], there is a lack of scientific information about sweat electrolyte losses in endurance specialties such as the marathon. In fact, previous recommendations about salt intake in the marathon are not completely based on scientific data obtained in marathoners [23]. In a marathon, the most frequent electrolyte imbalance is termed hyponatremia and this clinical condition is associated with serum sodium concentration below 135 mmol·L^−1^ [24, 25]. The prevalence of hyponatremia in marathon runners can be as high as 13 % [21]. Avoiding overhydration appears to be the most important means for preventing hyponatremia in the marathon [26, 27] and other endurance disciplines [28] but atypically high sweat Na^+^ losses have also been suggested as possible mechanisms to develop hyponatremia [3, 29, 30]. A recent investigation has determined that marathoners with atypical sweat Na^+^ concentrations (>60 mmol·L^−1^) presented lower serum electrolyte concentrations at the end of the race, although there were no cases of hyponatremia [14].

The aim of this investigation was to determine sweat electrolyte concentration in a large group of endurance runners participating in a real marathon. The other purpose of this investigation was to explain factors related to the high inter-individual variability found for sweat Na^+^ concentration. We hypothesized that sweat Na^+^ concentration would be predicted by individual characteristics such as body morphology and running pace.

## Methods

### Participants

A total of 157 healthy and experienced marathon runners (men = 141 and women = 16) volunteered to participate in this study. Before enrolling in the investigation, participants completed a questionnaire about previous training, running experience and previous best race time in the marathon. At this time, body mass and body height were measured (mod 284, Seca, Germany), while body mass index and body surface area [31] were calculated for each individual afterwards. Potential participants with a history of muscle disorders, cardiac or kidney disease or those taking medications were excluded. Age and main morphological and physical variables of participants in this investigation are shown in Table 1. Blood variables [14] and CFTR genotype [20] were measured in a subset of these participants and the results have been published elsewhere.

**Table 1:** Age, anthropometric characteristics, running performance, running experience and training status of the marathoners. Data is mean ± SD for each group

Variable (units)	Men	Women	*P* value
N	141	16	-
Age (y)	41.9 ± 9.7	42.0 ± 6.1	0.98
Body mass (kg)	75.0 ± 9.2	56.6 ± 7.1	<0.01
Body surface area (m^2^)	1.9 ± 0.1	1.6 ± 0.1	<0.01
Height (m)	176 ± 7	161 ± 5	<0.01
Body mass index (kg · m^−2^)	24.1 ± 2.2	20.9 ± 1.2	<0.01
Best race time (min)	217 ± 34	240 ± 32	0.03
Running experience (yr)	10.7 ± 9.8	9.5 ± 5.9	0.67
Completed marathons (number)	7 ± 3	5 ± 2	0.42
Average training distance · week^−1^ (km)	62.7 ± 27.8	59.5 ± 26.6	0.69
Training sessions of running · week^−1^ (number)	4.3 ± 1.0	4.4 ± 1.0	0.68

*P* values correspond to U Mann–Whitney tests used to compare differences between sexes

### Experimental design

A descriptive and comparative study, using an ecological experimental design, was used for this investigation. All the participants completed the 2014 edition of the Rock’n’Roll Madrid Marathon. The marathon race was held in April 2014 on a sunny day with 24.4 ± 3.6 °C of mean dry temperature and 27.7 ± 4.8 % of mean relative humidity. The day of the race, participants had their pre-competition meal at least 3 h before the race and were encouraged to drink 500 mL of plain water 2 h before the start of the race. Fifteen-to-thirty minutes before the race, participants arrived at an area close to the start line after their habitual warm-up, in their competition clothes and after they had emptied their bladders. At this time, pre-race body mass was measured (±50 g scale; Radwag, Poland) and two sweat patches (Tegaderm + Pad, 3 M, US) were placed on their left forearm to collect sweat samples during the race. For this measurement, the skin on the forearm was gently cleaned with alcohol and distilled water, and subsequently dried with clean gauze to eliminate any remains of previous sweat from the skin. The sweat patch was then firmly adhered to the skin and fastened by an elastic tubular net bandage (Elastofix, Insfarma, Germany).

Then, participants were directed to the start line and completed the race with no instructions about food or drinking and ran at their own pace. Participants drank *ad libitum* at the hydration stations placed at 5-km intervals. During the race, participants wore a race bib with a time-chip to calculate the net time employed to complete the race.

Within 2 min of the end of the marathon race, participants went to a finish area where body mass was immediately measured using the same apparatus previously described. Participants were instructed to avoid drinking from the finish line till the post-race weighing and a researcher assured compliance. At this time, the sweat patches were removed using clean tweezers and placed in a sterile 10 mL- tube. Sweat patches that were detached from the skin or presented a leak were discarded. Then, participants filled out a detailed questionnaire about fluid and food intake during the race. Data on this questionnaire was used to calculate fluid and electrolyte intake during the race using the nutritional facts of the products consumed and a nutrition software (PCN software, Cesnid, Spain). Participants were also asked about stops during the race to urinate or defecate. None of the participants reported any of these types of stops. Sweat loss volume (in L) was calculated as the pre-to-post-race change in body mass plus the amount of fluid and food ingested during the race. Sweat rate (in L·h^−1^) were calculated from sweat loss volume and race time. Relative sweat salt loss (in g per L of sweat) was calculated by multiplying sweat Na^+^ and Cl^−^ concentrations by their respective atomic mass and adding the resulting values. Total sweat salt loss (in g) during the race was calculated from relative sweat salt loss and sweat volume, considering that all the losses of sweat Na^+^ and Cl^−^ were in the form of sodium chloride (NaCl). All these calculations have been based on previous investigations carried out with athletes [1, 10, 32–34].

### Sweat samples analysis

The sweat was separated from the patches *in situ* by centrifugation (10 min at 5000 g), transferred to 5-mL sealed tubes and refrigerated at 4 °C [35]. Within 48 h after the race, sweat osmolality was measured with a freezing point osmometer (model 3320, Advanced Instrument, MA) while sweat Na^+^, Cl^−^ and potassium (K^+^) concentrations were measured by duplicate using photoelectric flame photometry (Cobas 6000, Roche, IN). Sweat K^+^ values comparable to serum K^+^ concentrations provided evidence that electrolyte leaching from the epidermal layer was minimum [36].

### Statistical analysis

The variables were initially checked for normality using the Shapiro-Wilk test. The comparison between male and female marathon runners was performed by using U-Mann–Whitney tests for unpaired samples. After a preliminary analysis, the study sample was divided into three groups according to their sweat Na^+^ concentration obtained during the marathon. Participants with sweat Na^+^ concentration lower than 30 mmol·L^−1^ were classified as low-salt sweaters, participants with sweat Na^+^ concentration between 30 and 60 mmol·L^−1^ were considered to be typical sweaters, and participants with sweat Na^+^ concentration higher than 60 mmol·L^−1^ were considered to be salty sweaters. These three groups were established based on previous investigations that consider sweat sodium concentration as “high” when it reaches > 60 mmol·L^−1^ [23, 34]. The comparison between these three groups (low-salt, typical and salty sweaters) was performed by using one way analysis of variance (ANOVA; including the Tukey *post-hoc)* for quantitative variables and by using Chi square tests for qualitative variables. The relationship between variables was assessed by Pearson’s correlation coefficient. For each significant difference found in this study, we have calculated the effect size (ES) proposed by Cohen [37]. The data was analysed with the statistical package SPSS version 19.0 (SPSS Inc., Chicago, IL). The significance level was set at *P* < 0.05. Data is presented as mean ± standard deviation (SD) for each group of participants. In addition, the range (minimum value – maximum value) has been included for each sweat variable.

## Results

As a group, sweat Na^+^ concentration was 42.9 ± 18.7 mmol·L^−1^ (7.0 – 95.5 mmol·L^−1^), sweat Cl^−^ concentration was 32.2 ± 15.6 mmol·L^−1^ (7.3 – 90.6 mmol·L^−1^) and sweat K^+^ concentration was 6.0 ± 0.9 mmol·L^−1^ (3.1 – 8.0 mmol·L^−1^). Sweat osmolality was 146.2 ± 52.6 mOsm · kg H_2_O^−1^ (55.0 – 286.0 mOsm · kg H_2_O^−1^). Interestingly, women presented lower sweat Na^+^ (33.9 ± 12.1 *vs* 44.0 ± 19.1 mmol·l^−1^; *P* = 0.05, ES = 0.52) and Cl^−^ (22.9 ± 10.5 *vs* 33.2 ± 15.8 mmol·L^−1^; *P* = 0.01, ES = 0.65) concentrations than men, with no significant differences in sweat K^+^ concentration (6.4 ± 1.0 *vs* 6.0 ± 0.9 mmol·L^−1^; *P* = 0.10) or sweat osmolality (129.1 ± 41.0 *vs* 149.3 ± 55.2 mmol·L^−1^; *P* = 0.16). Women also presented a lower sweat rate than men (0.5 ± 0.2 vs 1.0 ± 0.3 L · h^−1^; *P* = 0.01, ES = 1.67) and a lower running pace during the race (2.8 ± 0.4 vs 3.1 ± 0.5 m · s^−1^; *P* = 0.04, ES = 0.66).

**Fig. 1:**
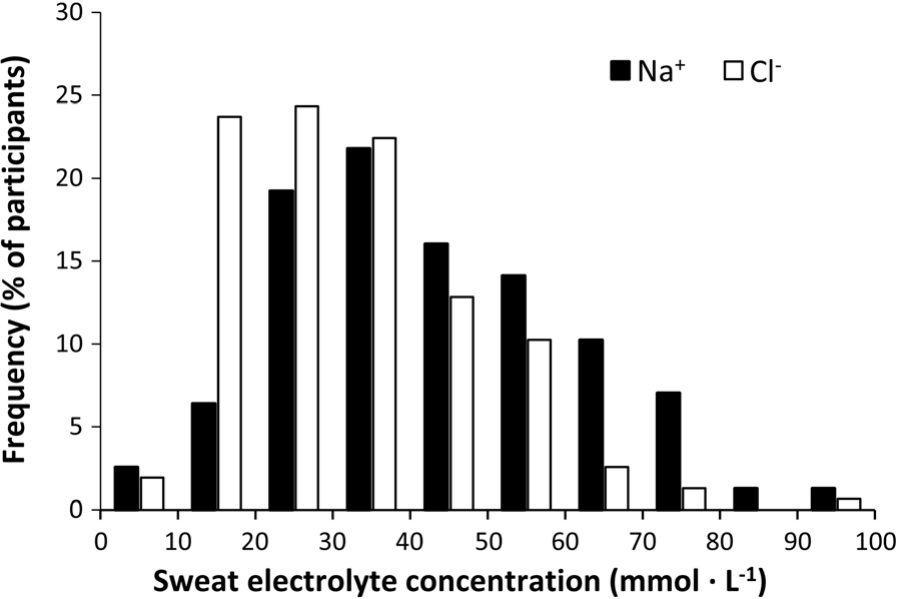
Frequency marathoners according to their sweat Na^+^ and sweat Cl^−^ concentrations. Data corresponds to 157 runners (141 men and 16 women) competing in a marathon

Figure 1 shows the frequency of marathoners according to their sweat Na^+^ and Cl^−^ concentrations to show the high inter-individual variability of these variables. The study sample presented a normal distribution for these sweat electrolyte concentrations and percentile 85 was 63 mmol·L^−1^ for sweat Na^+^ and 43 mmol·L^−1^ for sweat Cl^−^ concentrations. By using increasing mathematical ranges of 30 mmol·L^−1^, it was established that 26.8 % of the study sample can be classified as low-salt sweaters, 53.5 % of the sample as typical sweaters and 19.7 % of the sample as salty sweaters. The group of salty sweaters was entirely composed of male marathoners while the remaining two groups included women participants. Low-salt sweaters showed inferior concentrations of sweat Na^+^ (ES = 3.1 and 7.8, respectively) and Cl^−^ concentration (ES = 2.3 and 8.7, respectively), and lower sweat osmolality (ES = 1.3 and 2.6, respectively) than typical and salty sweaters (*P* < 0.05; Table 2). However, these individuals presented similar sweat K^+^ concentration, sweat rate, running pace and previous training volume than their counterparts. Similarly, salty sweaters presented significantly higher sweat Na^+^ and Cl^−^ concentrations and sweat osmolality than the typical and low-salt sweaters (*P* < 0.05) with no differences in the sweat K^+^ concentration, sweat rate, running pace or previous training volume respect to the remaining groups (Table 2; all comparison with *P* > 0.05). The higher sweat Na^+^ and Cl^−^ concentration during the race produced a higher sweat salt (e.g., NaCl) wasting in the group of salty sweaters (Table 2). Fluid intake rate was very similar in all the three groups included in this investigation (*P* = 0.85). Besides, the amount of Na^+^, Cl^−^ and K^+^ ingested by means of food and drinks during the race were similar among groups (Table 2; all comparison with *P* > 0.05).

**Table 2:** Sweat and performance variables according to sweat electrolyte concentration. Data is mean ± SD for each group

Variable (units)	Low-salt sweat	Typical Sweat	Salty sweat	*P* value
	<30 mmol·L^−1^ [sweat Na^+^]	30–60 mmol·L^−1^ [sweat Na^+^]	>60 mmol·L^−1^ [sweat Na^+^]	
Number/Frequency	42/27 %	84/54 %	31/20 %	-
Men/women	37/5	73/11	31/0	0.11
Sweat Na^+^ concentration (mmol·l^−1^)	21.4 ± 6.4*	43.2 ± 8.8	71.0 ± 9.0*†	<0.01
Sweat Cl^−^ concentration (mmol·l^−1^)	16.4 ± 4.3*	31.9 ± 9.9	54.4 ± 10.7*†	<0.01
Sweat K^+^ concentration (mmol·l^−1^)	5.9 ± 0.9	5.9 ± 0.9	6.2 ± 0.6	0.26
Sweat osmolality (mOsm·kg H_2_O^−1^)	100.8 ± 38.9*	150.4 ± 42.1	206.3 ± 40.3*†	<0.01
Sweat rate (L·h^−1^)	0.9 ± 0.2	0.9 ± 0.3	1.0 ± 0.2	0.76
Sweat NaCl loss (g L^−1^)	1.1 ± 0.3*	2.1 ± 0.5	3.5 ± 0.6*†	<0.01
Fluid intake (L·h^−1^)	0.32 ± 0.18	0.31 ± 0.17	0.33 ± 0.15	0.85
Na^+^ intake (mmol)	12.3 ± 8.1	11.9 ± 9.9	14.6 ± 16.6	0.81
Cl^−^ intake (mmol)	21.3 ± 13.0	20.8 ± 15.0	24.0 ± 25.0	0.72
K^+^ intake (mmol)	5.1 ± 6.0	4.7 ± 7.9	6.0 ± 5.6	0.85
Running pace (m·s^−1^)	3.0 ± 0.4	3.1 ± 0.5	3.2 ± 0.5	0.18
Average training distance·week^−1^ (km)	65.1 ± 32.0	60.5 ± 26.7	64.2 ± 24.1	0.65

*P* values correspond to Chi square tests -used to identify differences for the ratio men/women- and ANOVA tests -used to establish among-groups differences for the remaining variables-. (*) Different from typical sweat at *P* < 0.05; (†) Different from low-salt sweat at *P* < 0.05

**Fig. 2:**
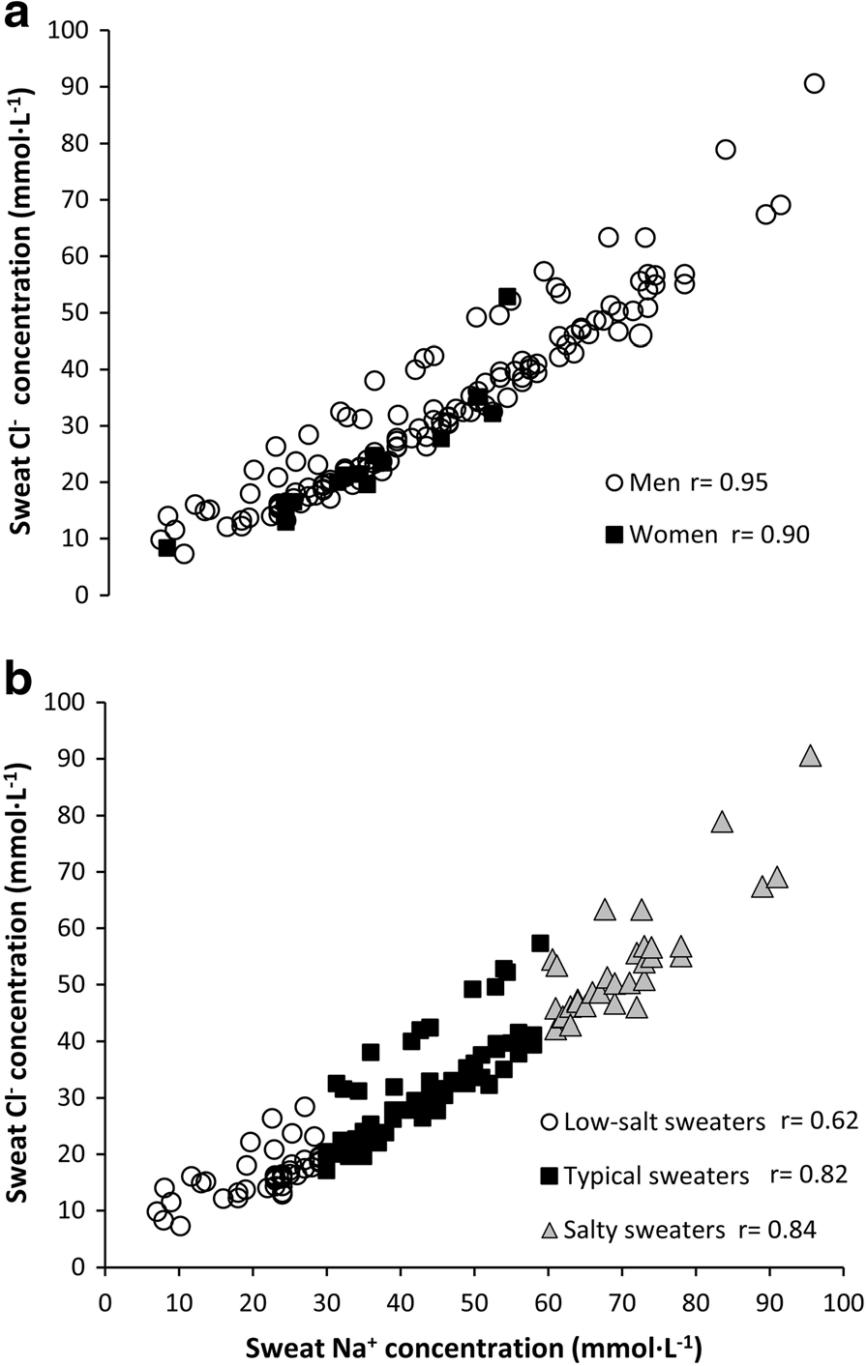
Relationship between sweat Na^+^ and sweat Cl^−^ concentrations in a group of marathoners. Panel **a** includes data organized by sex (141 men and 16 women). Panel **b** includes data of low-salty sweaters (<30 mmol·L^−1^ of sweat sodium concentration), typical sweaters (≥30 and < 60 mmol·L^−1^ of sweat sodium concentration) and salty sweaters (≥60 mmol·L^−1^ of sweat sodium concentration)

Sweat Na^+^ concentration did not significantly correlate to sweat rate, age, body characteristics (e.g., body mass, height, body mass index, body surface area), or training variables (experience, average training distance, training sessions per week). However, there was a weak but significant negative correlation between sweat Na^+^ concentration and running pace during the race (r = 0.18; *P* = 0.03). Sweat Cl^−^ did not significantly correlate to sweat rate, age, body characteristics, or training variables but it negatively correlated to running pace during the race (r = 0.18; *P* = 0.03). There was a strong and linear relationship between sweat Na^+^ and Cl^−^ concentrations indicating that both Na^+^ and Cl^−^ reabsorptions are strongly linked (Fig. 2).

## Discussion

It is well established that athletes of endurance disciplines are more prone to suffer electrolyte imbalances than athletes of shorter disciplines or team sports players, mainly because the differences in the duration of the sport competitions produce high differences in the total volume of sweat lost. [3] Nevertheless, there is limited information about sweat electrolyte concentration in endurance or ultraendurance competitions while the amount of data on sweat Na^+^ loss in team sports, with durations typically shorter than 90 min, is abundant [10, 38]. For this reason, the aim of this investigation was to determine sweat electrolyte concentration in a large group of endurance runners participating in a real marathon. The main outcomes of this investigation were: a) sweat Na^+^ and Cl^−^ concentrations in the marathon varied significantly among runners (Fig. 1) indicating that estimated sweat salt loss during the race showed great inter-individual variability. While most runners presented sweat Na^+^ concentrations below 60 mmol·L^−1^, 20 % of marathon runners presented atypically high sweat salt losses (e.g., salty sweaters, Table 2). b) sweat Na^+^ and Cl^−^ concentrations were lower in women than in men while there were no female runners classified as salty sweaters. The lower sweat electrolyte concentration in women coincided with a lower sweat rate, possibly allowing a greater reabsorption of electrolytes during their passage throughout the sweat gland duct [16]. However, other biological determinants of exercise thermoregulation, such as lower body mass and higher surface area-to-mass ratio [39], lower cholinergic stimulation of sweating [40] or differences in sex-specific hormones [27, 41] could have influenced the differences in sweat electrolyte concentration between sexes. c) sweat Na^+^ and Cl concentrations were not related to individual sweat rate, age, body characteristics or training variables but presented a weak correlation to running pace (r = 0.18; *P* = 0.03). All this information suggests the adequacy of measuring sweat electrolyte concentration to determine individual guidelines for salt intake during endurance disciplines. Universal recommendations [23] can underestimate the need for salt of a considerable proportion of endurance runners (e.g., salty sweaters).

During exercise, the secretory portion of the sweat gland produces a precursor fluid with a tonicity similar to the plasma upon cholinergic stimulation in an attempt to eliminate metabolic heat and to maintain body temperature homeostasis [42]. During its passage through the sweat duct the solutes of this precursor fluid are partially reabsorbed until producing a final sweat at the skin level, that is hypotonic respect to the plasma in healthy individuals [43]. Despite isolating factors that contribute to sweat solute retention such as acclimatization, training status and collection techniques, the excreted sweat greatly varies between individuals [44]. Because plasma or serum electrolyte concentration is very similar in healthy individuals [19], the inter-individual variability in sweat electrolyte concentration must be related to the solutes retention within the sweat duct [45].

Sweat ducts are impermeable to Na^+^ and Cl^−^ solutes but the CFTR protein acts as a cAMP-activated Cl^−^ channel to reabsorb Cl^−^ from the sweat. The CFTR protein also influences other membrane transport proteins such as epithelial Na^+^ channels (ENaC) [46] to reabsorb Na^+^ from sweat. In fact, a lower abundance of CFTR channels in the sweat duct limits both Na^+^ and Cl^−^ reabsorption and produces excretion of sweat with atypically high sweat electrolyte concentrations [43]. Figure 2 depicts individual data of sweat Na^+^ and Cl^−^ concentrations. Interestingly, there was a strong and linear relationship between sweat Na^+^ and Cl^−^ concentrations in all individuals, indicating that both Na^+^ and Cl^−^ reabsorptions are strongly linked. Moreover, this relationship was not modified when comparing men and women (panel A) or when comparing low-salt, typical and salty sweaters (panel B). This relationship suggests that, in salty sweaters, both Na^+^ and Cl^−^ retentions within the duct were less effective than in the remaining group of runners. Thus, excessive salt wasting by thermoregulatory sweat might be related to a low abundance of electrolyte channels in the membrane of the sweat duct or a dysfunctional in the CFTR protein [20]. An alternative explanation for the high sweat electrolyte concentration in healthy salty sweaters is low plasma concentration of aldosterone [47] and vasopressin [48], due to their actions to reabsorb sodium and water in the sweat gland. However, previous investigations have determined that these hormones do not explain inter-individual variability in sweat electrolyte losses [48] and consequently, they were not likely responsible for atypically high sweat electrolyte concentration.

Rehydration and salt intake during exercise aims to reduce water and electrolyte deficits caused by thermoregulatory sweating [44]. In most sports (<90 min of duration), the use of commercially available sports drinks to prevent body mass reductions > 2 % is an effective strategy to prevent water and electrolyte imbalances because the amount of sweat produced is not enough to challenge body (serum) electrolyte homeostasis [6, 8–10, 13, 49]. However, during endurance and ultra-endurance activities (>90 min of duration), especially in the heat, the amount of salt lost by thermoregulatory sweat can be very high, especially in salty sweaters. The main risk factors for the development of electrolyte imbalances during endurance disciplines, such as exercise-associated hyponatremia, are related to weight gain due to overdrinking prior or during exercise, exercise duration > 4 h and inexperience or inadequate training [27]. However, the influence of sweat salt concentration has been less considered as a cause for the development of electrolyte imbalances likely due to the fact that sweat electrolyte concentration has been rarely measured during endurance events.

A recent investigation has found that salty sweaters (>60 mmol·L^−1^ of sweat Na^+^ concentration) presented lower serum Na^+^ concentration and lower serum osmolality at the end of a marathon despite similar body mass loss and rehydration routines than typical or low-salt sweaters [14]. In that investigation, all marathoners rehydrated with commercially available beverages –water and sports drinks- which contain moderate concentrations of Na^+^ and salt (<30 mmol·L^−1^; < 1.7 of salt · L^−1^, respectively) [2]. While sport drinks can be an effective strategy to prevent electrolyte imbalance in most runners (e.g., low-salt and typical sweaters), the exclusive use of these beverages during the marathon was insufficient to avoid a reduction of serum electrolyte concentration in salty sweaters, likely due to the gap between sweat and beverage Na^+^ concentrations [14]. On the other hand, it has been found that “extra” sodium intake by way of using salt capsules -in addition to the rehydration routines with water and sport drinks- to achieve a replacement of 71 % of the salt lost by sweat during a half-ironman triathlon was an effective strategy to increase serum electrolyte concentration at the end of the race [1]. Thus, it is necessary to confirm in future investigations whether salty sweaters are more prone to suffering electrolyte imbalances in endurance activities and to establish if they might benefit from “extra” salt intake, beyond the amounts obtained with sports drinks.

While the strengths of the ecological experimental design used for this investigation have been discussed, it is also important to take the limitations into account to understand the outcomes of the present data. Firstly, due to the high sample size recruited and the experimental setting, a real marathon competition, we were unable to obtain post-race blood samples. Thus, we cannot conclude whether sweat salt wasting during the marathon due to atypically high sweat electrolyte concentration affected serum electrolyte homeostasis. A second limitation was related to the sample size of women marathoners that volunteered for this investigation. Although our sample (90/10 %) is representative of the ratio men/women runners in the 2014 Rock’n’Roll Madrid Marathon [50], the differences between male and female runners in sweat electrolyte concentration should be confirmed with a higher statistical power in the female group. Thus, all the information regarding sweating response in the sample of female marathoners should be interpreted with caution. A third limitation is the use of only two sweat patches on the forearm to collect sweat. Although this sweat collection procedure has been considered valid for the determination of sweat electrolyte concentration [4, 10, 35, 49], regional patch collection can slightly overestimate sweat electrolyte concentration when compared to the gold-standard procedure, whole body wash-down [51].

## Conclusions

Sweat Na^+^ and Cl^−^ concentrations are highly variable among marathoners (Fig. 1). Sweat salt loss cannot be truly predicted or estimated by individual characteristics or previous training, and thus the measurement of sweat rate and sweat electrolyte concentration is advised in order to improve the accuracy of salt intake recommendations during endurance activities. According to our data in amateur marathoners, it could be expected that ~80 % of runners presented sweat Na^+^ concentrations below 60 mmol·L^−1^. However, ~20 % of amateur marathon runners could be classified as “salty sweaters” with sweat salt losses equivalent to 3.5 ± 0.6 g of NaCl per litre of sweat. This means that an amateur runner with salty sweat, a race time of ~4 h and a sweat rate of ~1 L h^−1^ can loss ~14 g of NaCl during the whole race. It is necessary to investigate whether this salt wasting present in salty sweaters affects body electrolyte homeostasis in endurance disciplines as recently suggested [14].

## Abbreviations

ANOVA: Analysis of variance
cAMP: Cyclic adenosine monophosphate
CFTR: Cystic fibrosis transmembrane conductance regulator
Cl^−^: Chloride
ENaC: Epithelial channels for sodium
ES: Effect size
K^+^: Potassium
Na^+^: Sodium
NaCl: Salt or sodium chloride
SD: Standard deviation
